# Impact of early heparin therapy on outcomes in patients with solid malignancy associated sepsis: a marginal structural model causal analyse

**DOI:** 10.3389/fphar.2023.1281235

**Published:** 2023-12-04

**Authors:** Jia-jia Huang, Ji-zhen Cai, Zhi-peng Zhou, Yan Liu, Zhen-jia Yang, Da-zheng Li, Yu-hua Chen, Ying-yi Luan, Yong-ming Yao, Ming Wu

**Affiliations:** ^1^ Department of Infection and Critical Care Medicine, Shenzhen Second People’s Hospital and First Affiliated Hospital of Shenzhen University, Health Science Center, Shenzhen, China; ^2^ Intensive Care Unit, Shenzhen People’s Hospital (the Second Clinical Medical College, Jinan University; The First Affiliated Hospital, Southern University of Science and Technology), Shenzhen, China; ^3^ Department of Critical Care Medicine and Hematology, Xiangya Hospital, Central South University, Changsha, China; ^4^ Department of Nosocomial Infection Prevention and Control, Shenzhen Second People’s Hospital, Shenzhen, China; ^5^ Department of Emergency Medicine, Shenzhen Second People’s Hospital, Shenzhen, China; ^6^ Department of Central Laboratory, Beijing Obstetrics and Gynecology Hospital, Capital Medical University, Beijing, China; ^7^ Trauma Research Center, Medical Innovation Research Department and Fourth Medical Center of the Chinese PLA General Hospital, Beijing, China

**Keywords:** sepsis, heparin, mortality, thrombosis, solid malignancy

## Abstract

**Background:** Previous studies documented that heparin can inhibit the invasion and metastasis of tumors, but its role on outcomes in patients with solid malignancy complicated sepsis remains unclear.

**Methods:** A retrospective cohort study was conducted in critically ill patients with solid malignancy associated sepsis from the Medical Information Mart for Intensive Care (MIMIC)-IV database. The primary endpoint was intensive care unit (ICU) mortality, secondary outcomes were thrombosis and hospital mortality. Propensity score matching (PSM), marginal structural Cox model (MSCM), cox proportional hazards model, stratification analysis and E-value were used to account for baseline differences, time-varying confounding and unmeasured variables.

**Results:** A total of 1,512 patients with solid malignancy complicated sepsis were enrolled, of which 683 in the heparin group with intensive care unit mortality, thrombosis rate and hospital mortality were 9.7%, 5.4%, 16.1%, and 829 in the non-heparin group with ICU mortality, thrombosis rate and hospital mortality were 14.6%, 12.5%, 22.6%. Similar results were observed on outcomes for patients with PSM (ICU mortality hazard ratio [HR] 0.61, 95% confidence interval [CI] 0.41–0.92), thrombosis rate (HR 0.42, 95% confidence interval 0.26–0.68); hospital mortality HR 0.70, 95% CI 0.50–0.99). marginal structural Cox model further reinforced the efficacy of heparin in reducing ICU mortality (HR 0.48, 95% CI 0.34–0.68). Logistic regression and Cox regression model showed heparin use also markedly reduced thrombosis (HR 0.42; 95% CI 0.26–0.68; p < 0.001) and hospital mortality (HR 0.70; 95% CI 0.50–0.99; p = 0.043). Stratification analysis with the MSCM showed an effect only those with digestive system cancer (HR 0.33, 95% CI 0.16–0.69).

**Conclusion:** Early heparin therapy improved outcomes in critically ill patients with solid malignancy complicated sepsis. These results are evident especially in those with digestive system cancer. A prospective randomized controlled study should be designed to further assess the relevant findings.

## Introduction

Over the past decades, although clinical research on tumor therapy has improved rapidly, but cancer is the leading cause of deaths in all over the world (Bray et al., 2021; Sung et al., 2021). Targeted antitumor therapy mainly affects tumor formation and regulation, the tumor microenvironment, specific tumor markers, immune modulators, and targeted tumor stem cells (Danai et al., 2006; Vincent et al., 2009; Schünemann et al., 2020; Khan et al., 2021). Antitumor therapy readily releases damage-associated molecules in patients with solid malignancies, exacerbating undesirable inflammatory responses (Yuan et al., 2020). Many tumor-targeting drugs may affect angiogenesis, damage endothelial cells, and lead to cancer-associated coagulopathy, which increases the risk of thrombosis. It has been demonstrated that patients with cancer are more likely to develop venous thromboembolism (VTE) than those without cancer (Schünemann et al., 2020).

Patients with cancer exhibit an increased risk of sepsis owing to immune dysfunction. It is reported that, among cancer cases, the sepsis incidence increases by approximately 10 times than that of non-cancer cases (Vincent et al., 2009). A recent study showed that malignant tumors can be detected in 1/6 of intensive care unit (ICUs) inpatients experiencing sepsis (Danai et al., 2006), and if solid tumors patients suffer sepsis shock, the 28-day mortality rate was 69.4% (Cuenca et al., 2022). With the development of social economy and the improvement of clinical treatment, patients with cancer are always admitted to the ICU because of secondary sepsis and sepsis-induced coagulopathy (SIC). SIC and cancer-associated coagulopathy intensify VTE incidence among patients with solid malignancies concomitant sepsis.

As an anticoagulation agent, heparin has been widely used for prophylaxis VTE for several decades. Previous studies documented that heparin inhibited the invasion and metastasis of tumors (Wei et al., 2023), but limited data exists on whether heparin administration provide a survival advantage in patients with solid malignancies concomitant sepsis. Therefore, we conducted a retrospective cohort study adopted data based on the Medical Information Mart for Intensive Care IV (MIMIC-IV) database to assess whether anticoagulation with heparin used in solid malignancy concomitant sepsis cases hospitalized in the ICU offered survival benefits, including advantages in ICU mortality, hospital mortality, and thrombosis, and estimated heparin application timing as well as dosing.

## Materials and methods

### Data source and study design

We performed a retrospective cohort study using data from MIMIC-IV (version 1.0). It included two in-hospital database systems, namely, ICU-specific clinical data and the custom hospital-wide electronic health record (EHR), which covered anonymous, integrated clinical information of cases at ICUs in Beth Israel Deaconess Medical Center in Boston, Massachusetts, from 2008 to 2019. Individuals finishing a collaborative institutional training initiative examination (certification number 38995627 for author Huang) were allowed to enter this database.

### Participants

Altogether 382,278 individuals with 51,150 patients were admitted to the ICUs during our research. Patient selection criteria were as follows: 1) those aged over 18 years, 2) those meeting Sepsis 3.0 criteria (namely, a rapid elevation of Sequential Organ Failure Assessment (SOFA) score ≥2 in combination with suspicious infection (Singer et al., 2016), and 3) those diagnosed with malignant cancer.

Patients conforming to the following conditions were excluded: 1) those admitted to the ICU several times; 2) those aged <18 years or stayed in the ICU for <24 h; 3) those who used heparin in treatment or dialysis, but not in prophylactic application, or those who received warfarin or low-molecular-weight heparin (LMWH) in ICU stay; 4) pregnant women; 5) those with previous heparin-caused thrombocytopenia; 6) those with liver failure; 7) those with stage 5 chronic kidney disease (CKD); and 8) those with hematological malignancies.

### Research procedures and definitions

The study collected information based on the MIMIC-IV database by Structured Query Language. Approaches described previously were adopted to search the above database (sepsis) and analyze the patient information collected (Zou et al., 2022). For patients who were hospitalized repeatedly, initial hospitalizations were collected. Basic features, together with laboratory findings on day one upon ICU admission, were extracted, which included age upon admission to the hospital, gender, weight, laboratory examinations (hemoglobin levels, white blood cell [WBC] count, platelet count, international normalized ratio [INR], prothrombin time [PT], partial thromboplastin time [PTT]), vital signs (heart rate, temperature, respiratory rate, mean arterial pressure [MAP], partial pressure of oxygen [PO_2_]), concurrent diseases (diabetes, hypertension, chronic lung disease, chronic heart disease (CHD)), urine output, mechanical ventilation, vasopressor use, acute kidney injury (AKI) stage, and renal replacement therapy (RRT). This study further collected the results of clinical severity scales, such as the SOFA score or Simplified Acute Physiology Score II (SAPS II). Typically, the SOFA score was determined in the initial 24-h after post-admission to the ICU. AKI diagnosis was based on the Kidney Disease: Improving Global Outcomes (KDIGO) standards (Ostermann et al., 2020). AKI stages were determined based on creatinine and urine output levels within the initial 24-h after post-admission to the ICU.

The laboratory variable activated partial thromboplastin time (APTT) was extracted throughout all ICU stay. In addition, the present work obtained the physiological values and measurement chart time based on a database. If the cases were measured repeatedly, only the highest daily APTT was included in the analyses. These screening variables had <15% missing values (Supplementary Table S1) and were later subjected to single imputation.

### Exposure and outcomes

Participants were divided into two groups: a heparin group that enrolled patients receiving subcutaneous heparin 24 h after post-admission into the ICU at preventive doses and a control group that included patients not receiving heparin on day one. Our primary outcome was ICU mortality, which was deemed to be patient survival upon discharge from the ICU. The secondary outcomes included in-hospital mortality and thrombosis.

### Statistical analysis

Categorical variables were presented in the form of numbers and percentages. They were compared between the heparin and nonheparin groups using Fisher’s exact test and chi-square test, whereas continuous variables were expressed as mean (standard deviation, SD) or median (interquartile range [IQR]) as appropriate.

The present study adopted propensity score matching (PSM) to interpret the basic differences in whether the patient received heparin treatment (Huang et al., 2023). During PSM analysis, prophylactic heparin was administered to the heparin group 24 h post-ICU admission. The treatment group was then matched to the control group through nearest-neighbor matching. Besides, this work also determined standardized mean difference (SMD) was calculated to examine whether PSM reduced the differences between two groups. Finally, a Cox proportional hazards model was used to adjust for residual imbalance by including parameters with *p* values < 0.05 and potential confounding factors judged by clinical expertise.

In this study, we determined heparin administration during the ICU stay to be the time-dependent variable for the marginal structural Cox model (MSCM). The possible basic confounders, including age, gender, mechanical ventilation, vasopressor, RRT, SOFA, and SAPS II scores, were acquired on the first day after ICM entry. The APTT in the entire ICU stay period was a time-varying confounder and was incorporated into the above model. In addition, MSCM parameters were predicted based on inverse probability weighting (IPW) (IPW) to correct selection bias or confounders, such as informative censoring (Shinozaki and Suzuki, 2020). IPW was adopted to weigh all cases, which helped to create two pseudopopulations close in terms of basic and time-dependent confounders, whereas they were distinct with regard to heparin exposure. IPWs were estimated using the IPW package (Grafféo et al., 2019).

To explore the differences in heparin use and ICU mortality among diverse subgroups stratified according to gender, race, AKI stage, vasopressor medication, mechanical ventilation use, and malignant cancer, a stratification analysis was performed. The Cox model, with adjustments for every variable in basic patient characteristics, was adopted for the subgroup analysis. E-values were determined to analyze the probability of non-measured confounders of heparin with ICU mortality (Haneuse et al., 2019). E-values could be used to quantify the necessary magnitude of the non-methylated confounder to negate the detected relationship of heparin with ICU mortality. Additionally, a pre-specified subgroup analysis was carried out using MSCM. *p* < 0.05 (two-tailed) stood for statistical difference. R software (version 4.1.1) was used for the analysis.

## Results

### Patient characteristics

**FIGURE 1:**
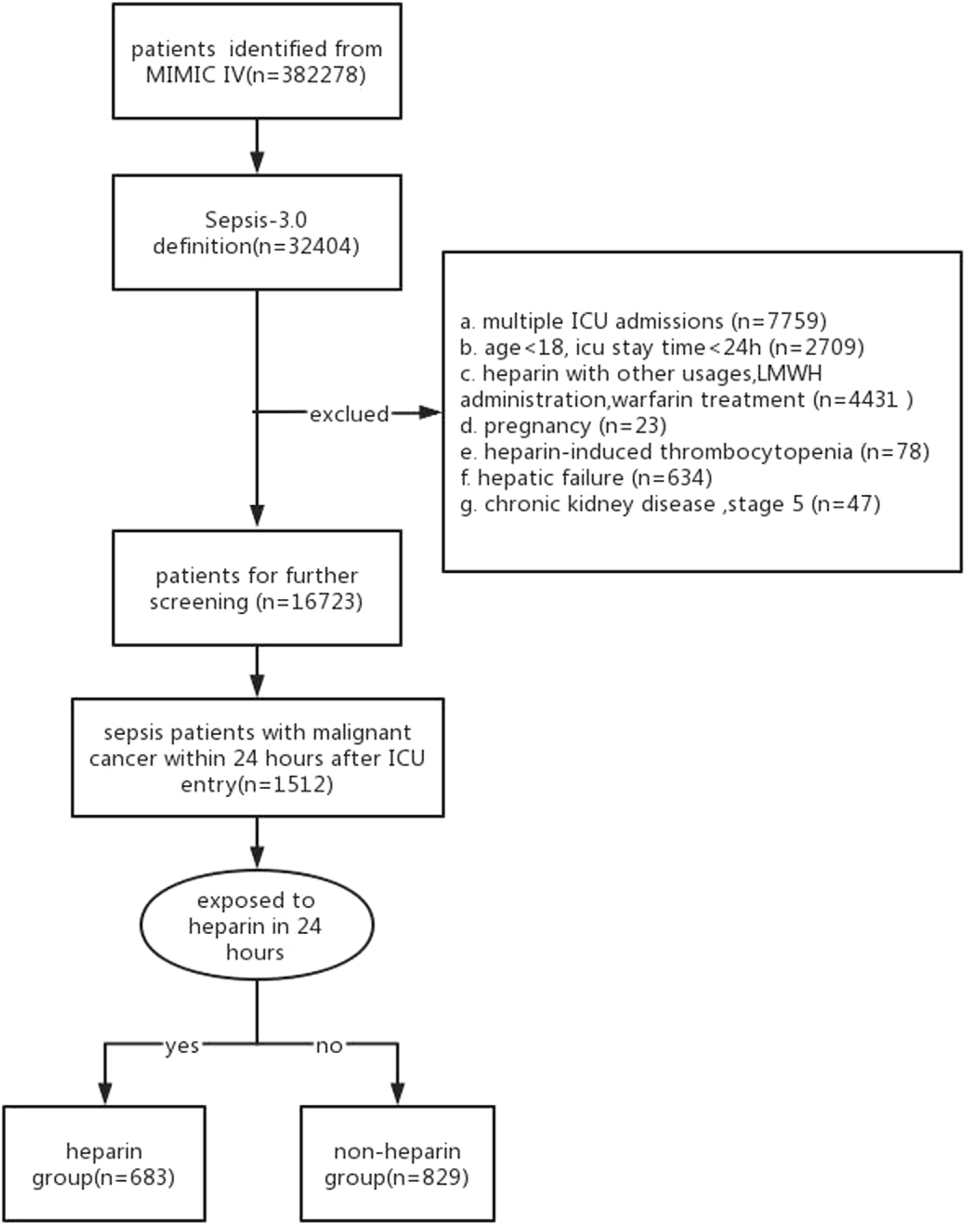
Flowchart of participants selection by the Sepsis-3 definition.

Upon a preliminary search, 382,278 ICU entries were found in the MIMIC-IV database. There were 32,404 cases satisfying the definition, while 1,512 septic patients had complicated malignant cancer 24 h after admission into the ICU. Of those study cases, 683 received heparin treatment within the initial 24 h post-admission to the ICU, whereas the remaining 829 did not administer heparin (Figure 1).

As shown in Table 1, temperature, hemoglobin levels, platelet levels, INR, PT, presence of diabetes, presence of chronic heart disease, AKI stage, ventilation, and SOFA score were significantly different between the two groups. Notably, the non-heparin group had a higher number of critical cases than the heparin group (SOFA score, 6 [4.8] vs. 5 [3.8], *p* < 0.001). Moreover, the heparin group displayed an increased requirement for mechanical ventilation (38.9% vs. 33.2%, *p =* 0.023) compared to the non-heparin group.

**TABLE 1:** Baseline characteristics of critically ill patients with solid malignancy associated sepsis before and after propensity score matching.

	Propensity score matching							
	Before				After			
Patients characteristics	No heparin (n = 829)	Heparin (n = 683)	*p*-value	SMD	No heparin (n = 528)	Heparin (n = 528)	*p*-value	SMD
**Demographics**								
Gender, male (%)	539 (65.0)	414 (60.6)	0.087	0.091	329 (62.3)	337 (63.8)	0.655	0.031
Age (yr)	68.97 (12.97)	68.89 (12.70)	0.909	0.006	68.77 (13.41)	68.71 (12.98)	0.948	0.004
Ethnicity (%)			0.305	0.136			0.946	0.08
White	567 (68.4)	472 (69.1)			366 (69.3)	366 (69.3)		
Asian	43 (5.2)	41 (6.0)			32 (6.1)	34 (6.4)		
Black	89 (10.7)	65 (9.5)			42 (8.0)	49 (9.3)		
Other	130 (15.7)	105 (15.4)			88 (16.6)	79 (15.0)		
Weight (kg)	78.08 (19.95)	78.14 (21.21)	0.953	0.003	77.41 (20.24)	77.89 (21.21)	0.706	0.023
Heart rate (bpm)	89.37 (16.98)	89.13 (16.04)	0.775	0.015	89.32 (17.18)	89.79 (16.35)	0.648	0.028
MAP (mmHg)	76.22 (10.08)	75.48 (10.05)	0.157	0.073	75.85 (9.62)	75.80 (10.28)	0.938	0.005
Respiratory rate (bpm)	19.79 (4.22)	19.77 (4.14)	0.956	0.003	19.55 (4.31)	19.87 (4.25)	0.23	0.074
Temperature (°C)	36.84 (0.46)	36.89 (0.49)	0.031	0.111	36.86 (0.47)	36.86 (0.48)	0.891	0.008
Spo2 (%)	90.79 (7.64)	91.27 (5.68)	0.174	0.071	91.38 (7.20)	90.93 (6.09)	0.27	0.068
**Laboratory findings**								
Hemoglobin (g/L)	9.18 (2.13)	9.84 (1.89)	<0.001	0.328	9.72 (2.08)	9.68 (1.88)	0.736	0.021
Minimum platelet (10³/μl)	173 (116)	212 (114)	<0.001	0.339	201 (118)	197 (109)	0.506	0.041
WBC (10³/μl)	15.32 (12.10)	15.25 (9.96)	0.897	0.007	15.05 (10.00)	15.33 (10.36)	0.651	0.028
Maximum INR	1.73 (1.13)	1.47 (0.46)	<0.001	0.302	1.48 (0.49)	1.50 (0.48)	0.508	0.041
PT(s)	18.67 (11.55)	16.09 (4.81)	<0.001	0.291	16.17 (5.16)	16.39 (5.03)	0.47	0.044
APTT(s)	37.01 (19.44)	37.47 (16.71)	0.628	0.025	36.94 (21.37)	38.20 (17.25)	0.293	0.065
**Complication**								
Hepertension,n (%)	337 (40.7)	309 (45.2)	0.081	0.093	228 (43.2)	222 (42.0)	0.756	0.023
Diabetes,n (%)	109 (13.1)	177 (25.9)	<0.001	0.326	97 (18.4)	108 (20.5)	0.437	0.053
Chronic heart disease,n (%)	85 (10.3)	100 (14.6)	0.012	0.133	62 (11.7)	66 (12.5)	0.777	0.023
Chronic pulmonary disease,n (%)	221 (26.7)	187 (27.4)	0.798	0.016	134 (25.4)	137 (25.9)	0.888	0.013
Urine output (mL)	1,679 (1,231)	1,617 (1,046)	0.299	0.054	1,641 (1,214)	1,644 (1,060)	0.960	0.003
AKI stage,n (%)			0.023	0.159			0.983	0.025
0	420 (50.7)	303 (44.4)			246 (46.6)	246 (46.6)		
1	121 (14.6)	97 (14.2)			78 (14.8)	76 (14.4)		
2	186 (22.4)	198 (29.0)			142 (26.9)	140 (26.5)		
3	102 (12.3)	85 (12.4)			62 (11.7)	66 (12.5)		
RRT,n (%)	17 (2.1)	11 (1.6)	0.66	0.033	6 (1.1)	8 (1.5)	0.788	0.033
Vasopressor,n (%)	344 (41.5)	252 (36.9)	0.077	0.094	217 (41.1)	212 (40.2)	0.802	0.019
Ventilation,n (%)	275 (33.2)	266 (38.9)	0.023	0.12	207 (39.2)	193 (36.6)	0.41	0.055
SOFA score, median (IQR)	6 [4.8]	5 [3.8]	0.001	0.166	5 [4.8]	5 [3.75.8]	0.873	0.01
SAPS II score, median (IQR)	42 [32.57]	40 [32.50]	0.099	0.085	41 [33.50]	41 [32.50]	0.834	0.013

Abbreviations: MAP, mean arterial pressure; AKI, acute kidney injury; WBC, white blood cell; INR, international normalized ratio; PT, prothrombin time; APTT, activated partial thromboplastin time; RRT, renal replacement therapy; SOFA, sequential organ failure assessment; SAPS II, simplified acute; physiology score II., Values are shown as the mean (SD) unless otherwise indicated. n, IQR,interquartile range.

## Outcomes

### Propensity score analysis

A total of 1,512 solid malignancy patients with sepsis were enrolled in the study, of which 683 in the heparin group had ICU mortality, thrombosis rate, and hospital mortality of 9.7%, 5.4%, 16.1%, and 829 in the non-heparin group, and ICU mortality, thrombosis rate, and hospital mortality were 14.6%, 12.5%, and 22.6%, respectively (Table 2). PSM was then conducted to match 528 cases receiving heparin with 528 patients not receiving heparin, which markedly decreased the imbalances between both groups (Supplementary Figure S1, Table 1). Owing to the presence of residual imbalances between both groups, this study utilized the Cox proportional hazards model. As a result, heparin use led to decreased mortality for the entire cohort (ICU mortality: hazard ratio [HR],0.61; 95% CI 0.41–0.92; *p* = 0.019). Secondary outcomes were similar (hospital mortality: HR, 0.70; 95% CI 0.50–0.99; *p = 0.043,* thrombosis: HR:0.42; 95% CI 0.26–0.68; *p <* 0.001) (Table 2).

**TABLE 2:** Association between heparin use and clinic outcomes in critically ill patients with solid malignancy associated sepsis.

Outcomes n (%)	Propensity score matching cohort (n = 1,056)					All eligible for propensity score (n = 1,512)				
	All patients n = 1,056	Non-heparin n = 528	Heparin n = 528	HR (95%)	*p-*value	All patients n = 1,512	Non-heparin n = 829	Heparin n = 683	HR (95%CI)	*p*-Value
Primary										
ICU mortality	131 (12.4)	77 (14.6)	54 (10.2)	0.61 (0.41.0.92)	0.019	187 (12.4)	121 (14.6)	66 (9.7)	0.67 (0.46.0.97)	0.033
Secondary										
Thrombosis	87 (8.2)	60 (11.4)	27 (5.1)	0.42 (0.26.0.68)	<0.001	141 (9.3)	104 (12.5)	37 (5.4)	0.44 (0.30.0.66)	<0.001
Hospital mortality	206 (19.5)	115 (21.8)	91 (17.2)	0.70 (0.50.0.99)	0.043	297 (19.6)	187 (22.6)	110 (16.1)	0.66 (0.49.0.90)	0.008

Abbreviations: HR, hazard ratio; ICU, intensive care unit.

### Marginal structural cox model and stratification analysis

**FIGURE 2:**
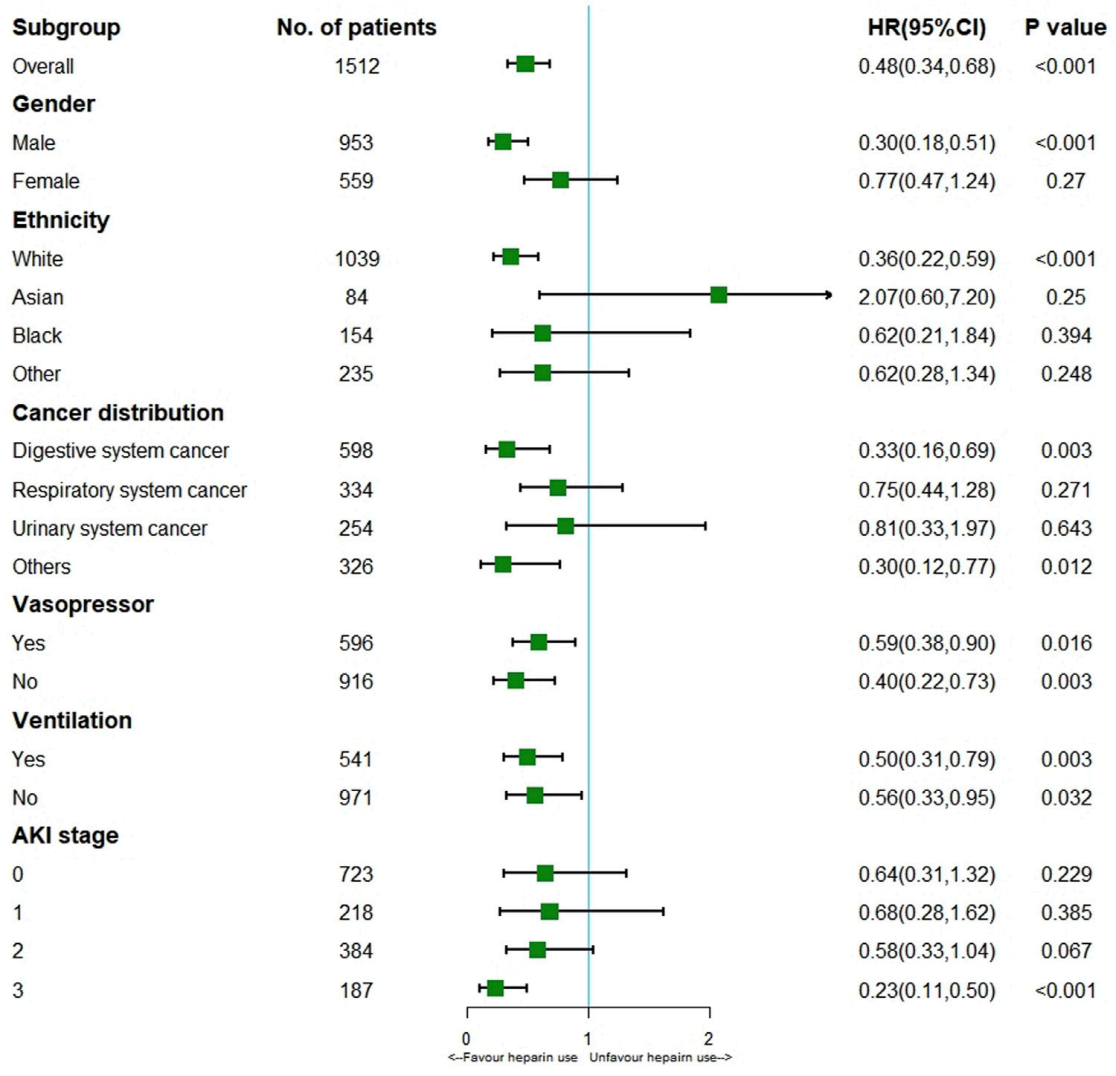
Results of ICU mortality in overall population with MSCM and stratification analysis Abbreviations: AKI, acute kidney injury; HR, hazard ratio.

This study incorporated heparin use and time-varying confounders into MSCM. As a result, heparin use reduced ICU mortality (HR 0.48; 95% CI 0.34–0.68; *p <* 0.001) for the entire cohort. According to the stratification analysis results, heparin use decreased the incidence of ICU mortality among patients with solid malignancies concomitant sepsis among males (HR 0.30; 95% CI 0.18–0.51; *p <* 0.001) and those of white ethnicity (HR 0.36; 95% CI 0.22–0.59; *p* < 0.001), stage 3 AKI (HR 0.23; 95% CI 0.11–0.50; *p* < 0.001), and digestive system cancer (HR 0.33; 95% CI 0.16–0.69; *p* = 0.003) (Figure 2). It was observed that, in patients with solid malignancy concomitant sepsis, treatment with 7,500–12500 IU heparin daily was associated with a lower risk of ICU mortality than no heparin treatment (Table 3).

**TABLE 3:** Dose-response relationship between heparin and ICU mortality in critically ill patients with solid malignancy associated sepsis.

Daily heparin usage (non-heparin group as References)	No. Of patients^a^	HR (95%CI)	*p*-Value
0U < *x* ≤ 5000U	174	1.33 (0.65.2.75)	0.433
5000U < *x* ≤ 7500U	217	0.61 (0.28.1.32)	0.210
7500U < *x* ≤ 10000U	266	0.47 (0.27.0.82)	0.008
10000U < *x* ≤ 12500U	121	0.25 (0.12.0.53)	<0.001
12500U < *x* ≤ 15000U	90	0.55 (0.22.1.39)	0.204

^a^ Number of patients receiving prophylactic heparin.

### Logistic regression, cox regression model and stratification analysis

**FIGURE 3:**
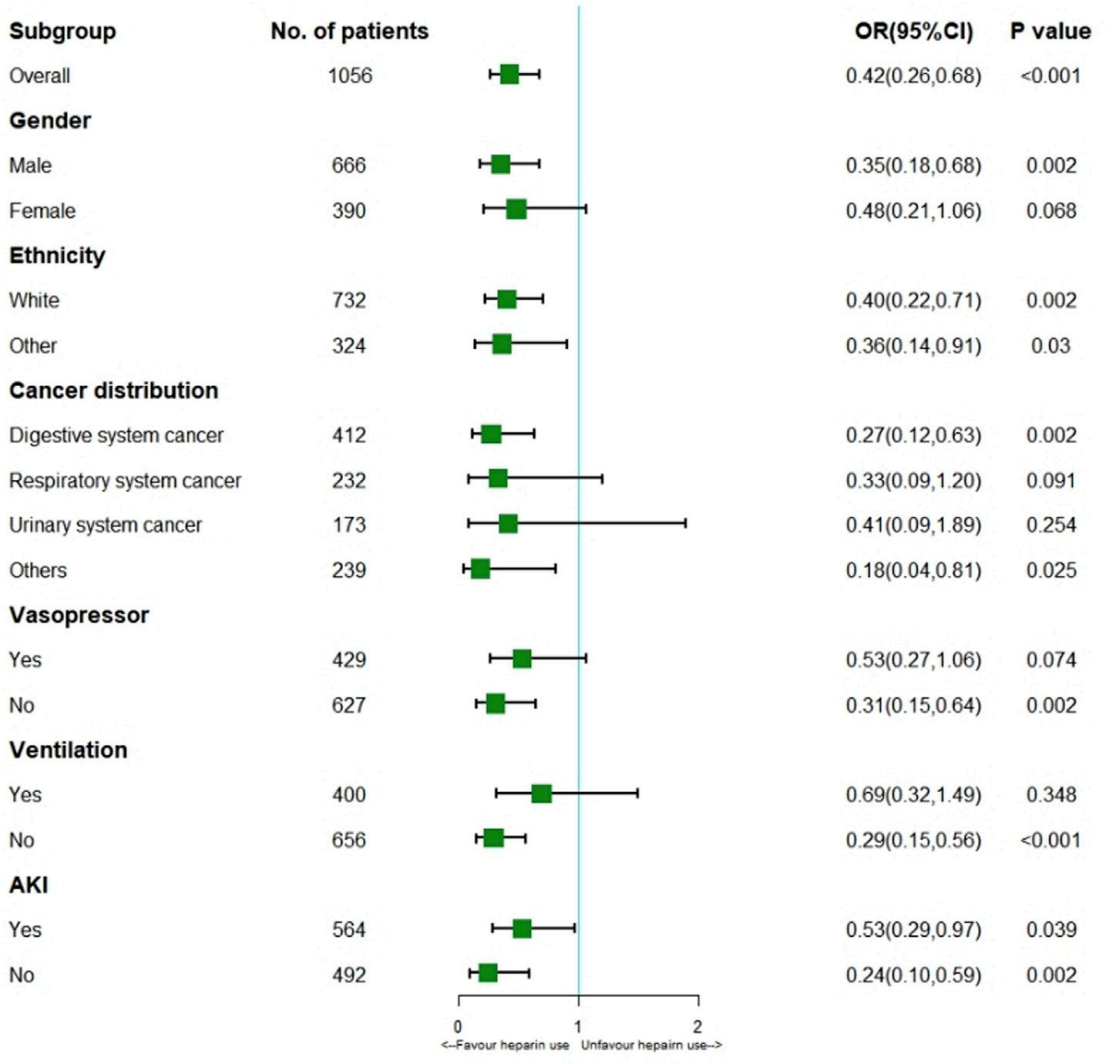
Results of thrombosis in overall population with logistic regression model and stratification analysis. Abbreviations: AKI, acute kidney injury; OR, odds ratio.

Logistic regression model showed heparin use markedly reduced thrombosis (HR 0.42; 95% CI 0.26–0.68; *p* < 0.001) for the entire cohort. According to the stratification analysis results, heparin use decreased the incidence of thrombosis in patients with solid malignancies concomitant sepsis among males (HR 0.35; 95% CI 0.18–0.68; *p* = 0.002) and those of white ethnicity (HR 0.40; 95% CI 0.22–0.71; *p* = 0.002) or other ethnicity (HR 0.36; 95% CI 0.14–0.91; *p* = 0.03), digestive system cancer (HR 0.27; 95% CI 0.12–0.63; *p* = 0.002), non-Vasopressor use (HR 0.31; 95% CI 0.15–0.64; *p* = 0.002), non-Ventilation (HR 0.29; 95% CI 0.15–0.56; *p* < 0.001) and AKI (HR 0.53; 95% CI 0.29–0.97; *p* = 0.039). (Figure 3).

**FIGURE 4:**
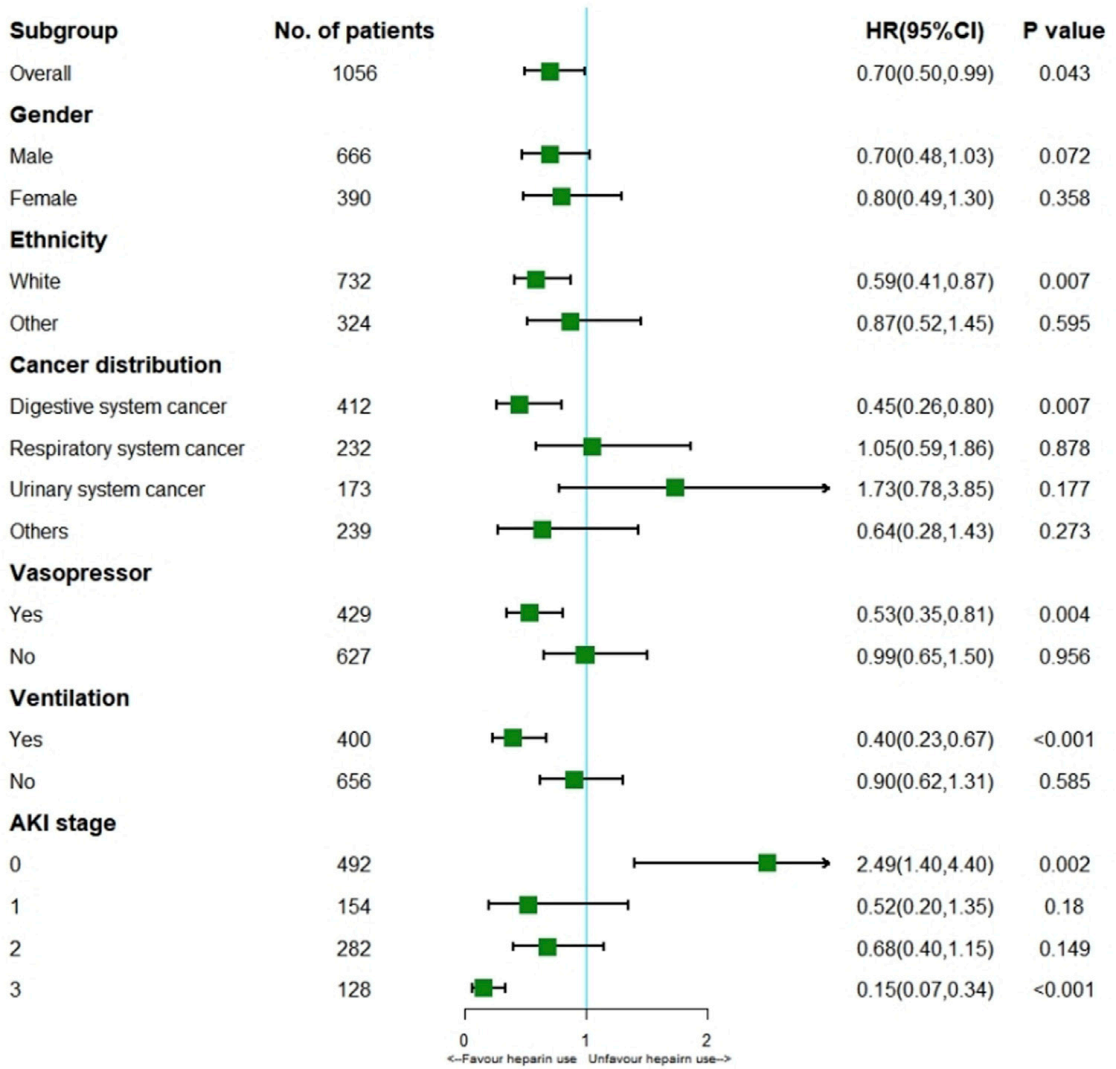
Results of hospital mortality in overall population with cox regression model and stratification analysis.

Cox regression model showed heparin use markedly reduced hospital mortality (HR 0.70; 95% CI 0.50–0.99; *p* = 0.043) for the all cohort. According to the stratification analysis results, heparin use decreased the incidence of hospital mortality in patients with solid malignancies concomitant sepsis among those of white ethnicity (HR 0.59; 95% CI 0.41–0.87; *p* = 0.007), digestive system cancer (HR 0.45; 95% CI 0.26–0.80; *p* = 0.007), Vasopressor use (HR 0.53; 95% CI 0.35–0.81; *p* = 0.004), Ventilation (HR 0.40; 95% CI 0.23–0.67; *p* < 0.001) and stage 3 AKI (HR 0.15; 95% CI 0.07–0.34; *p* < 0.001). (Figure 4).

### Sensitivity analysis

Distinct measured and known ICU mortality-related risk factors identified in the multivariable Cox proportional hazards model following PSM were SAPS II (HR, 1.02; 95% CI, 1.01–1.04), AKI 2 (HR, 1.91; 95% CI, 1.10–3.32), chronic pulmonary disease (HR, 2.27 (1.51.3.41); 95% CI, 1.51–3.41), and diabetes (HR, 1.74; 95% CI, 1.09–2.79) (Table 4).

**TABLE 4:** Multivariable cox regression model after propensity score matching in critically ill patients with solid malignancy associated sepsis.

Variables	HR (95%CI)	*p*
Gender		
Famale	References	
Male	0.96 (0.62.1.47)	0.850
Age	1.00 (0.98.1.01)	0.670
Weight	0.99 (0.98.1.00)	0.212
SPO_2_	0.96 (0.94.0.98)	<0.001
PT	0.84 (0.67.1.05)	0.122
SOFA	1.05 (0.96.1.13)	0.276
SAPS II	1.02 (1.01.1.04)	0.008
AKI stage		
0	References	
1	1.60 (0.84.3.02)	0.151
2	1.91 (1.10.3.32)	0.022
3	1.62 (0.76.3.47)	0.209
Vasopressor use		
No	References	
Yes	0.87 (0.54.1.40)	0.560
Ventilation use		
No	References	
Yes	0.55 (0.34.0.89)	0.016
Chronic heart disease		
No	References	
Yes	1.09 (0.57.2.07)	0.800
Chronic pulmonary disease		
No	References	
Yes	2.27 (1.51.3.41)	<0.001
Diabetes		
No	References	
Yes	1.74 (1.09.2.79)	0.021
Hypertension		
No	References	
Yes	0.85 (0.58.1.24)	0.386

Abbreviations: PT, prothrombin time; AKI, acute kidney injury; RRT, renal replacement therapy; SOFA, sequential organ failure assessment; SAPS II, Simplified Acute Physiology Score II.

E-values were analyzed to assess the sensitivity to non-measured confounders (https://www.evalue-calculator.com/evalue/). Reliable preliminary results were obtained, only if non-measured confounders existed, the ICU mortality risk was relatively low and the HR was over 2.66 (upper limit of 4.05), suggesting the capability of residual confounders of explaining the detected connection when one non-measured covariate with the relative risk association with prophylactic heparin use and ICU mortality of >2.66 existed. Therefore, unknown and non-measured confounders might not significantly affect ICU mortality (relative risk >2.66) compared with known risk factors.

## Discussion

Due to the immunosuppression observed in cancer patients, sepsis may develop. Although animal studies have demonstrated that heparin can combine with lipopolysaccharide (LPS) to reduce the mortality resulting from Gram-negative bacterial infection (Tang et al., 2021; Yuan et al., 2021), and we found that early administration heparin provide a survival advantage in critically ill patients with sepsis (Zou et al., 2022), unfortunately, cancer patients were excluded from our previous studies. Thus, this conclusion may not be suitable for patients with cancer complicated with sepsis. Notably, minimal data exist on anticoagulation with heparin on outcomes in critically ill patients with solid malignancy associated sepsis as far. Whether administration heparin would provide a survival advantage in critically ill patients with solid malignancies concomitant sepsis remains unknown. Therefore, we designed another study on effectiveness of heparin therapy to patients with solid malignancies, and the results from the MIMIC-IV data demonstrate that heparin administration is associated with improved ICU mortality, thrombosis and hospital mortality in patients with solid malignancy concomitant sepsis.

As administration heparin is time-dependence variable, so we employed MSCM model to adjust additional time-dependent interventions (de Keyser et al., 2014; Karim et al., 2014; Huang et al., 2023). The MSCM further reinforces the efficacy of heparin in reducing ICU mortality. For the unmeasured confounding variables, we used risk factor analysis with a multivariable Cox proportional hazards model and E-value analysis to perform a combined analysis of the data. The result indicates that it is unlikely that an unmeasured confounder would have a substantially greater effect on ICU mortality than these known risk factors. E-value analysis suggested robustness to unmeasured confounding variables (Haneuse et al., 2019). Therefore, heparin therapy did demonstrate a benefit in patients not only in general populations but also in those with solid malignancies. Interestingly, stratification analysis with MSCM further indicated that administration heparin at 7,500–12500 IU a day decreased ICU mortality only among male patients and those with white ethnicity, stage 3 AKI, digestive system cancer and did not benefit those with respiratory system cancer. The underlying mechanism may involve the patient’s ethnicity and race, endocrine metabolism status, tumor type and targeted therapy. The findings presented in this report provide an insight role of heparin in patients with solid malignancies concomitant sepsis.

During targeted therapy for patients with solid malignancies, injury-related molecules are readily released and trigger inflammatory reactions and coagulation disorders (Wu et al., 2021), leading to an increased risk of thrombosis. Patients with cancer are significantly more likely to develop venous thromboembolism (VTE) than people without cancer (Heit, 2015; Schünemann et al., 2020). Cancer-associated thrombosis is the second leading cause of death in cancer patients after disease progression (Farge et al., 2022), furthermore, the incidence of cancer-associated thrombosis is increasing worldwide (Schünemann et al., 2020). The prevalence of cancer-associated thrombosis is increasing because of multiple factors, including longer patient survival, the use of anticancer therapies, increased detection of incidental VTE during surveillance imaging and wider use of central venous catheters (Mulder et al., 2021). The annual risk of a venous thromboembolic event in patients with solid cancer is 4%–5% overall, with wide variation between patients with different cancer types (Akl et al., 2017). A systematic review identified the greatest benefit from heparin treatment for VTE in patients with lung cancer (RR 0.59, 95% CI 0.42–0.81) (Cuenca et al., 2022). The result is similar to our results that heparin treatment has a benefit on reducing the risk of thrombosis (HR 0.42, 95% CI 0.26–0.68, *p* < 0.001). Therefore, the international clinical practice guidelines included the recommendation of LMWHs for the initial (first 10 days) treatment and maintenance treatment of cancer-associated thrombosis (Farge et al., 2022), but it does not improve survival (Cuenca et al., 2022).

Although previous studies have documented that heparin can inhibit tumor invasion and metastasis (Wei et al., 2023), heparin therapy has not been used as a conventional antitumor method in clinical practice. Recently, in the United States, an observational cohort study based on over 1 million sepsis hospitalizations showed that in-hospital mortality in cancer-related sepsis patients was 27.9% vs*.* 19.5% in patients without cancer-related sepsis, and cancer-related sepsis was associated with an adjusted absolute increase in in-hospital mortality that ranged from 2.2% to 15.2% of that associated with noncancer-related sepsis (Hensley et al., 2019). There are many plausible explanations for the differential outcomes between cancer-related and noncancer-related sepsis, including the cancer itself, cancer treatment and the resulting immune suppression, critical care provider bias and the differing goals of care (Hensley et al., 2019). Research shows that approximately 70% of cancer patients in the ICU have solid malignancies, and there is no recommendation in the international sepsis guidelines on whether they need anticoagulation treatment (Evans et al., 2021). The results of this study will provide a new therapeutic insight for the treatment of solid malignancies associated sepsis. The mechanism of effectiveness of heparin therapy to patients with solid malignancies concomitant sepsis, apart from anticoagulation effect, anti-inflammatory effects, anticomplement activity, and protease regulation on heparin (Li et al., 2013; Li et al., 2014), the underlying mechanism still needs further to investigate.

However, certain limitations of the present study should be noted. First, this study was carried out based on an EHR using clinically derived data. Therefore, the process adopted in cohort screening may not be identical to guideline-defined sepsis. However, sepsis cases were identified based on guidelines identical to the third definition of sepsis (such as infection combined with rapid alteration of total SOFA score ≥2 points). Second, confounders might have affected our results because of the retrospective nature of the present work. As a result, MSCM and PSM were adopted for balancing critical confounders, both of which verified our result creditability. Finally, certain patient variables were not obtained from the database, which might have led to bias or confounders. E-values were calculated in the sensitivity analysis to quantify possible results caused by those non-extracted confounders; according to our results, the non-extracted confounders might not interpret the whole therapeutic effect.

## Conclusion

According to our results, early heparin application for patients with solid malignancy associated sepsis may improve survival, including ICU mortality, hospital mortality, and thrombosis, particularly for males and those with white ethnicity, stage 3 AKI, and digestive system cancer. In addition, patients with solid malignancy associated sepsis who received 7,500–12500 IU per day had lower ICU mortality than non-heparin cases. Consequently, more prospective randomized controlled trials are warranted to verify timing, dosage, and indications of heparin use for solid tumor cases.

## Data Availability

The original contributions presented in the study are included in the article/Supplementary Material, further inquiries can be directed to the corresponding authors.
