# Supplementary material for: Impact of early heparin therapy on outcomes in patients with solid malignancy associated sepsis: a marginal structural model causal analyse
Source: Front Pharmacol. 2023 Dec 4;14:1281235. doi: 10.3389/fphar.2023.1281235 (PMC10729002; doi:10.3389/fphar.2023.1281235)

***Supplementary materials***

Table S1. Missing number (%) for included variables in the dataset

| **Variables** | Missing (%) |
| --- | --- |
| Age | 0 (0) |
| Gender | 0 (0) |
| Weight | 30 (2.0%) |
| WBC | 4 (0.3%) |
| Hemoglobin | 4 (0.3%) |
| Minimum platelet | 4 (0.3%) |
| Maximum INR | 145 (9.6%) |
| APTT | 152 (10.1%) |
| PT | 145 (9.6%) |
| SPO_2_ | 0(0) |
| Temperature | 18 (1.2%) |
| MAP | 0(0) |
| Heart rate | 0 (0) |
| Respiratory rate | 0 (0) |
| Hypertension | 0 (0) |
| Diabetes | 0 (0) |
| Chronic heart disease | 0 (0) |
| Chronic pulmonary disease | 0 (0) |
| Vasopressor | 0 (0) |
| Mechanical ventilation | 0 (0) |
| RRT | 0 (0) |
| SOFA | 0 (0) |
| SAPSII | 0 (0) |
| Urine output | 14 (0.9%) |
| AKI stage | 12 (0.8%) |
| Thrombin | 0 (0) |
| ICU mortality | 0 (0) |
| Hospital mortality | 0 (0) |
| Length of ICU stay | 0 (0) |
| Length of hospital stay | 0 (0) |

Abbreviations: WBC, white blood cell; INR, international normalized ratio; PT, prothrombin time; APTT, activated partial thromboplastin time; MAP, mean arterial pressure; AKI, acute kidney injury; RRT, renal replacement therapy; SIC, sepsis-induced coagulopathy; SOFA, sequential organ failure assessment; SAPS II, simplified acute physiology score II.

Fig. S1. Standardized mean difference (SMD) of variables before and after propensity score matching.


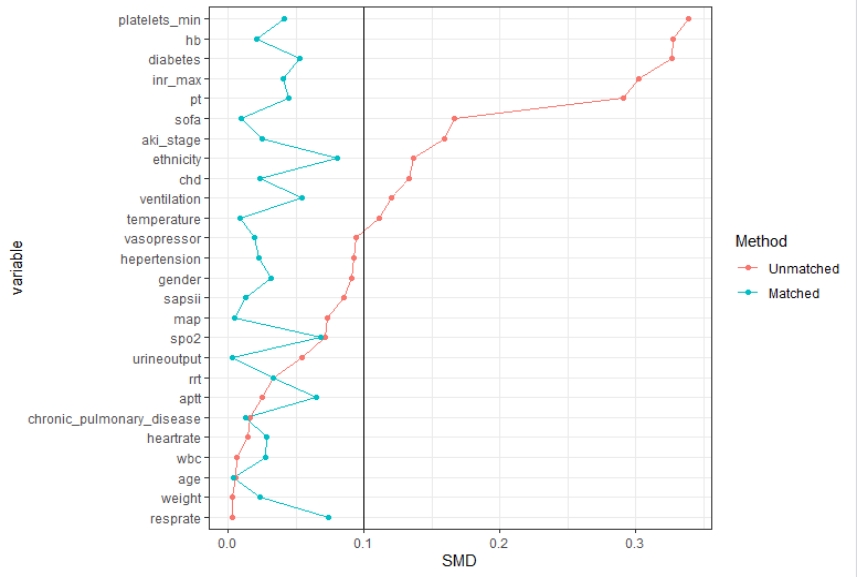

Supplement: Supplementary file 1 [file DataSheet1.docx]
